# Editorial: Quality of life improvement: smart approaches for the working and aging populations

**DOI:** 10.3389/fpubh.2024.1362019

**Published:** 2024-03-11

**Authors:** Sabina Baraković, Zahid Akhtar, Jasmina Baraković Husić

**Affiliations:** ^1^Faculty of Traffic and Communications, University of Sarajevo, Sarajevo, Bosnia and Herzegovina; ^2^Department of Network and Computer Security, State University of New York Polytechnic Institute, Utica, NY, United States; ^3^Faculty of Electrical Engineering, University of Sarajevo, Sarajevo, Bosnia and Herzegovina

**Keywords:** aging, quality, life, smart, working

## Introduction

Quality of life (QoL) is a notion that can be defined and treated in very complex manners depending on the context. It can be seen as a perception of a person's life and satisfaction and overall enjoyment with it, as well as its wellbeing, which depends on many diverse factors stemming from various domains such as health, psychological state, security and safety, beliefs and meaning, social position, environment conditions, relationships, economy position, etc. (1).

European Framework 8+1 (2) categorizes those factors affecting Quality of life in the following eight groups: (i) material living conditions—income, consumption, and material conditions; (ii) health—healthy and unhealthy behaviors, and access to healthcare; (iii) education—competences and skills, lifelong learning, and opportunities for education; (iv) productive and valued activities—working, volunteering, etc.; (v) governance and basic rights—institutions and public services, discrimination and equal opportunities, and active citizenship; (vi) leisure and social interactions—quantity and quality of leisure, as well as access to leisure, and the social dimension; (vii) natural and living environment—pollution, access to green and recreation spaces, as well as landscape and built environment; and (viii) economic and physical safety—wealth, debt, and income insecurity from the economic side, and crime and a perception of physical safety from the physical side. Lastly, it includes overall experience of life as an important influent as well.

Improving Quality of life as a multidimensional and complex concept should be a goal of research activities in general for all population groups (children, teenagers, young adults, parents, women, men, unemployed, employed, older adults, etc.). However, it is especially interesting to address it in terms of working and aging populations. Namely, demographic data suggests an increased need for workers worldwide and a rapid aging trend in the active workforce and in general also. This trend of workforce deficit and population aging will be even more prominent in the future (3). Statistics given by United Nations (4) say that by 2050 there will be 1.6 billion people aged 65 years or older (one in six people), while the number of people aged 80 years or older is growing even faster. Also, as fertility levels fall, the share of young people declines, while the shares of working-age adults and older people go up.

Therefore, if one wants to remain concurrent in the upcoming challenging economic environment with lack of workforce, increased costs, labor supply chain reduction, or successfully cope with difficulties related to the needs of older adults and their caregivers and family members (such as lack of care force, finances, etc.), smart approaches need to be developed and utilized for improving the Quality of life for working and aging population groups. Those smart approaches include policies, methods, and practices, as well as technologies and solutions. For example, those smart approaches could cover the use of technology and innovation such as Internet of things (IoT), Web of things (WoT), Virtual reality (VR), Augmented reality (AR), Artificial intelligence (AI), etc. in products, services, solutions, and systems in public and private concept used to improve the Quality of life of older adults and working population which is aging. In addition, the application of new, creative, original, and inventive ideas to policies and strategies can domain-depending step-by-step improve the working conditions by supporting, for example, private-business life balance, adjusting tasks to age, etc. thereby improving the Quality of workers life and extending their working ability.

## Smart approaches for Quality of life improvement of aging and working population

The Editorial aims to present the contributing articles of the Research Topic related to Smart Approaches for the Working and Aging Populations in terms of Quality of life Improvement. The Research Topic has published articles received from September 2022 to February 2023 regarding: (i) smart policies, methods, and practices, technologies, digital solutions, as well as security and privacy issues of technologies and solutions contributing to Quality of life; (ii) policies, methods, and practices, technologies and solutions, as well as security and privacy issues of technologies and solutions related to smart and healthy aging; (iii) policies, methods, and practices, technologies and solutions, as well as security and privacy issues of technologies and solutions related to smart and healthy working; and lastly (iv) artificial intelligence solutions related to Quality of life improvement, smart and healthy aging and working. These studies were conducted in countries worldwide which indicates that this topic is popular and contemporary in all corners of the world and it should be addressed by the research community more intensively.

The first group of articles refers to smart policies, methods, and practices, technologies, digital solutions, as well as security and privacy issues of technologies and solutions contributing to Quality of life. Four articles fit into this group presenting several technology solutions aimed at enhancing the Quality of life for older adults. Gambo et al. provided the review of current technological solutions designed for older adults, including the following aspects: work life, community engagement, and wellbeing at home. Although smart home technology holds promise in encouraging sustainable living, its adoption among older adults remains modest. Wei et al. explored how intergenerational relationships impact the willingness of seniors to accept smart home services and found positive impact on empowering aging individuals to continue living independently by leveraging technology. As global unemployment rises, young generations are turning to the informal sector for employment opportunities, where the high risk of occupational hazards calls for effective healthcare. Therefore, Oladosu et al. identified existing factors affecting access to healthcare in order to clarify the pathways by which they may impact the health and Quality of life of young generations, providing insights to guide policy development. In similar study, Grakh et al. identified stressors and stress levels among veterinary students in order to assist in developing and implementing coping strategies to safeguard the mental health of students, contributing thereby to their Quality of life.

The second group of articles refers to policies, methods, and practices, technologies and solutions, as well as security and privacy issues of technologies and solutions related to smart and healthy aging. Nine articles can be classified into this group summarizing recent research on given topic. The conventional model of older adults care services faces challenges, including the outdated design of information platforms, subpar quality in caregiving, and issues related to the “digital divide.” Consequently, Zhang and Xu proposed an enhancement in the quality of older adults care services through the implementation of a smart older adults care service model, rooted in grassroots medical and health care. Moreover, Wang and Zhu examined the importance of constructing public health informatization, with a specific emphasis on the components involved in such construction. Furthermore, Zhao et al. found that the smart medical system has the potential to offer significant convenience to both the older adults and community healthcare. In that sense, Rmadi et al. evaluated head-mounted display virtual reality tolerance among older nursing home residents through cybersickness and anxiety state. Abdulrazak et al. proposed approach utilizes unobtrusive IoT technology and algorithms for detecting change points to monitor the daily health status of older adults. Jiang and Liu studied how does the community- and home-based medical care service affect the social participation of the chronically ill older adults. Sun discussed an establishment of the emergency material reserve mechanism for public health emergencies and optimization of the management of various functional departments while proposing to strengthen management personnel allocation and optimize work processes. Additionally, Wang, Liu, Zheng, et al. concluded that comprehensive feedback mechanism for older adults can mitigate speculative practices by providers of older adults care services and platforms, and government can dynamically adjust penalties and subsidy policies. Finaly, when it comes to policies, Zhang, Ning et al. proposed three policy recommendations to promote the development of a new model of integration of sports and medicine in China.

The third group of articles includes seven studies and refers to policies, methods, and practices, technologies and solutions, as well as security and privacy issues of technologies and solutions related to smart and healthy working. In this context, Wang, Liu, Zhu, et al. made an investigation into enhancing the occupational safety and employment safeguards for takeout workers within the framework of public health optimization. Kalski et al. focused on preventive health examinations in order to early identify health-related risk factors and maintain workability. Zhang, Zhang et al. explored the present condition of health insurance governance for full population coverage in China along with its influencing factors, and offered empirical insight that can be referenced by countries sharing similar social backgrounds with China. Furthermore, Qin et al. explored the influence of job satisfaction and sleep quality on the correlation between work stress and depression among Chinese healthcare workers, and whether the mediation models varied based on differences in educational degrees. Alif et al. identified a decline in disability-free survival among individuals engaged in “elementary” occupations, particularly those entailing elevated accident risks and unfavorable social climates. Chen et al. provided evidence supporting the idea that the combination of low wages and extended working hours constitutes notable occupational factors that have an adverse effect on the subjective sleep quality of female care workers from Southeast Asia employed in Taiwan. Lastly, Zhu et al. implemented tailored psychological adjustments and interventions with the aim to safeguard the mental wellbeing of civil servants, enhance their proficiency in addressing public emergencies, and empower them to apply accurate and positive psychological approaches when dealing with emergencies and high-pressure situations.

In the end, the fourth group of articles refers to artificial intelligence solutions related to Quality of life improvement, smart and healthy aging and working. In this sense, Gu et al. proposed the evaluation model of express service for addressing the shortcomings of the service industry and enhancing the quality of service.

## Conclusion

Considering all of the above, this Editorial can provide the following conclusions. Although it covers research studies on policies, methods, and practices, technologies and digital solutions contributing to Quality of life, as well as smart and healthy aging and working, associated security and privacy issues are not sufficiently addressed. In addition, artificial intelligence solutions related to Quality of life improvement, smart and healthy aging and working remain unexplored. These issues can serve as a starting point for future research activities, as well as idea for launching new Research Topics.

## Author contributions

SB: Writing – original draft, Writing – review & editing. ZA: Writing – original draft, Writing – review & editing. JBH: Writing – original draft, Writing – review & editing.
